# Suppressing disease spreading by using information diffusion on multiplex networks

**DOI:** 10.1038/srep29259

**Published:** 2016-07-06

**Authors:** Wei Wang, Quan-Hui Liu, Shi-Min Cai, Ming Tang, Lidia A. Braunstein, H. Eugene Stanley

**Affiliations:** 1 Web Sciences Center, University of Electronic Science and Technology of China, Chengdu 610054, China; 2 Big Data Research Center, University of Electronic Science and Technology of China, Chengdu 610054, China; 3 Center for Polymer Studies and Department of Physics, Boston University, Boston, Massachusetts 02215, USA; 4 Instituto de Investigaciones Físicas de Mar del Plata (IFIMAR)-Departamento de Física, Facultad de Ciencias Exactas y Naturales, Universidad Nacional de Mar del Plata-CONICET, Funes 3350, (7600) Mar del Plata, Argentina

## Abstract

Although there is always an interplay between the dynamics of information diffusion and disease spreading, the empirical research on the systemic coevolution mechanisms connecting these two spreading dynamics is still lacking. Here we investigate the coevolution mechanisms and dynamics between information and disease spreading by utilizing real data and a proposed spreading model on multiplex network. Our empirical analysis finds asymmetrical interactions between the information and disease spreading dynamics. Our results obtained from both the theoretical framework and extensive stochastic numerical simulations suggest that an information outbreak can be triggered in a communication network by its own spreading dynamics or by a disease outbreak on a contact network, but that the disease threshold is not affected by information spreading. Our key finding is that there is an optimal information transmission rate that markedly suppresses the disease spreading. We find that the time evolution of the dynamics in the proposed model qualitatively agrees with the real-world spreading processes at the optimal information transmission rate.

The coevolution dynamics on complex networks has attracted much attention in recent years, since dynamic processes, ubiquitous in the real world, are always interacting with each other^1^,^2^. In biological spreading dynamics, two strains of the same disease spread in the same population and interact through cross immunity^3^,^4^,^5^ or mutual reinforcement^6^. In social spreading dynamics, individuals are surrounded by multiple items of information supplied by, e.g., Facebook, Twitter, and YouTube. These sources of information compete with each other for the limited attention-span of users, and the outcome is that only a few items of information survive and become popular^7^,^8^. Recently scholars have become aware of the coevolution or interplay between biological and social spreadingdynamics^9^,^10^,^11^. When a new disease enters a population, if individuals who are aware of its potential spread take preventive measures to protect themselves^12^,^13^ the disease spreading may be suppressed. Our investigation of the intricate interplay between information and disease spreading is a specific example of disease-behavior systems^14^.

Studying the micromechanisms of a disease-behavior system can help us understand coevolution dynamics and enable us to develop ways of predicting and controlling the disease spreading^10^. In this effort a number of excellent models^15^,^16^,^17^ have demonstrated the existence of non-trivial phenomena that differ substantially from those when there is independent spreading dynamics^18^,^19^,^20^,^21^,^22^,^23^,^24^. Researchers have demonstrated that the outbreak of a disease has a metacritical point^16^ that is associated with information spreading dynamics and multiplex network topology and that information propagation is promoted by disease spreading^17^. Funk *et al*. found that the disease threshold is altered once the information and disease evolve simultaneously^15^. These models make assumptions about the coevolution mechanisms of information and disease spreading and do not demonstrate the interacting mechanisms in real-world systems. Because we do not understand the microscopic coevolution mechanisms between information and disease spreading dynamics from real-world disease-behavior systems, we do not have a systematic understanding of coevolution dynamics and do not know how to utilize information diffusion to more effectively suppress the spread of disease.

We present here a systematic investigation of the effects of interacting mechanisms on the coevolution processes of information and disease spreading dynamics. We first demonstrate the existence of asymmetrical interactions between the two dynamics by using real-world data from information and disease systems to analyze the coevolution. We then propose an asymmetric spreading dynamic model on multiplex networks to mimic the coupled spreading dynamics, which will allow us to understand the coevolution mechanics. The results, obtained from both the theoretical analyses and extensive simulations, suggest some interesting phenomena: the information outbreak can be triggered by its own spreading dynamics or the disease outbreak, while the disease threshold is not affected by the information spreading. Our most important finding is that there is an optimal information transmission rate at which the outbreak size of the disease reaches its minimum value, and the time evolution of the dynamics in the proposed model qualitatively agrees with the dynamics of real-world spreading.

## Results

### Empirical analysis of real-world coevolution data

Information about disease can be obtained in many ways, including face-to-face communication, Facebook, Twitter, and other online tools. Since the growth of the Internet, search engines have enabled anyone to obtain instantaneous information about disease. Patients seek out and analyze prescriptions using search engines in hopes of obtaining a means of rapid recovery. Healthy individuals use search engines to identify protective measures against disease to maintain their good health.

To examine the coevolution of real-world data about information and disease, we use weekly synchronously evolving data on information and disease systems associated with influenza-like illness (ILI) in the US during an approximate 200-week period from 3 January 2010 to 21 September 2013. The ILI dataset records weekly outpatient visits to medical facilities, and Google Flu Trends (GFT) dataset keeps track of week queries in Google search engine about ILI symptoms^25^. The GFT is used to analyse the occurrence probability of a disease^26^. For simplicity, we assume that the volume of information about the disease is proportional to the GFT volume because any individual can use the Google search engine to gain information about ILI. For a detailed description of the data see ref. 26.

**Figure 1:**
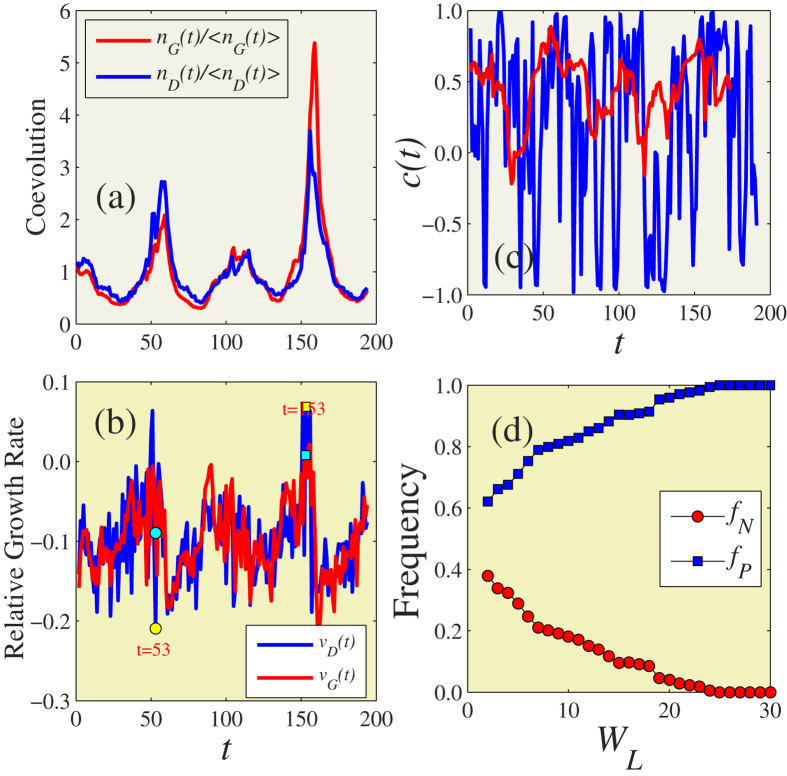
Weekly outpatient visits and Google Flu Trends (GFT) of influenza-like illness (ILI) from 3 January 2010 to and 21 September 2013 in the United States. (**a**) The relative number of outpatient visits *n*_*D*_(*t*)/〈*n*_*D*_(*t*)〉 (blue dashed line) and relative search queries aggregated in GFT *n*_*G*_(*t*)/〈*n*_*G*_(*t*)〉 (red solid line) versus *t*, where 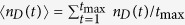 and 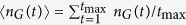, and *t*_max_ is the number of weeks. (**b**) The relative growth rate *v*_*D*_(*t*) (blue dashed line) and *v*_*G*_(*t*) (red solid line) of *n*_*D*_(*t*) and *n*_*G*_(*t*) versus *t*, respectively. (**c**) Cross-correlation *c*(*t*) between the two time series of *v*_*G*_(*t*) and *v*_*D*_(*t*) for the given window size *w*_*l*_ = 3 (blue dashed line) and *w*_*l*_ = 20 (red solid line). (**d**) The fraction of negative correlations *f*_*P*_ (blue squares) and positive correlations *f*_*N*_ (red circles) as a function of *w*_*l*_. In (**a**), *n*_*G*_(*t*) and *n*_*D*_(*t*) are divided their average values respectively. In (**b**), the circles and squares denote the relative growth rate at *t* = 53 and 153, respectively.

Figure 1(a) shows the real-data time series of information *n*_*G*_(*t*) and disease *n*_*D*_(*t*) indicating that macroscopically the two systems exhibit similar trends and confirming that the GFT effectively predicts disease spreading^26^,^27^ — although some researchers have expressed skepticism^28^. To identify the coevolution mechanisms operating between information and disease spreading, we further investigate the time series from a microscopic point of view. Specifically, we study their relative growth rates *v*_*G*_(*t*) of *n*_*G*_(*t*) and *v*_*D*_(*t*) of *n*_*D*_(*t*) (see definitions in Method Section). Figure 1(b) shows the evolution of *v*_*G*_(*t*) and *v*_*D*_(*t*). Note that the same and opposite growth trends of *v*_*G*_(*t*) and *v*_*D*_(*t*) coexist. For example, at week 53 (week 153), *v*_*G*_(53) > 0 [*v*_*G*_(153) > 0] and *v*_*D*_(53) < 0 [*v*_*D*_(153) > 0]. Thus the GFT and ILI show the opposite (the same) growth trends.

To conceptualize the correlations of the growth trends between the two dynamics, we analyze the cross-correlations *c*(*t*) between the time series of *v*_*G*_(*t*) and *v*_*D*_(*t*) for a given window size *w*_*l*_^29^ using the Pearson correlation coefficient *c*(*t*) between the two time series 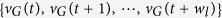 and 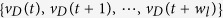. When *c*(*t*) > 0, the growth rates of information and disease share the same trend in the time interval *w*_*l*_. When *c*(*t*) < 0, the information and disease have opposite growth trends. Figure 1(c) shows that the positive and negative *c*(*t*) are uncovered for *w*_*l*_ = 3 and *w*_*l*_ = 20, respectively. This may be because individuals tend to search for disease information when they are infected or when someone they know is infected, and thus a disease outbreak promotes the spread of information, i.e., the growth trends of GFT and ILI will be the same. When individuals acquire information about the disease they then take action to protect themselves, and this causes the growth trends of GFT and ILI to go in opposite directions. We thus conclude that there are asymmetric interactions between the dynamics of information and disease spreading, i.e., disease spreading promotes information spreading, but information spreading suppresses disease spreading. Figure 1(d) plots the fraction of negative correlations *f*_*P*_ and positive correlations *f*_*N*_ as a function of *w*_*l*_. The fraction of positive correlations *f*_*P*_ (negative correlations *f*_*N*_) increases (decreases) with the *w*_*l*_, since individuals taking measures are dependent on the timeliness of the information. Note therefore that asymmetric interactions can only continue over a short period of time.

### Coevolution dynamics on multiplex networks

We now propose a novel model based on the coevolution mechanisms in real-world data, i.e., the asymmetric interactions between information and disease spreading. Information spreads through communication networks and disease usually spreads through contact networks. Communication and contact networks usually have different topologies. To describe the distinct transmission topologies of the information and disease we use a multiplex network^30^,^31^,^32^,^33^ and construct an artificial communication-contact coupled network without degree-degree correlations in intralayers and interlayers.

**Figure 2:**
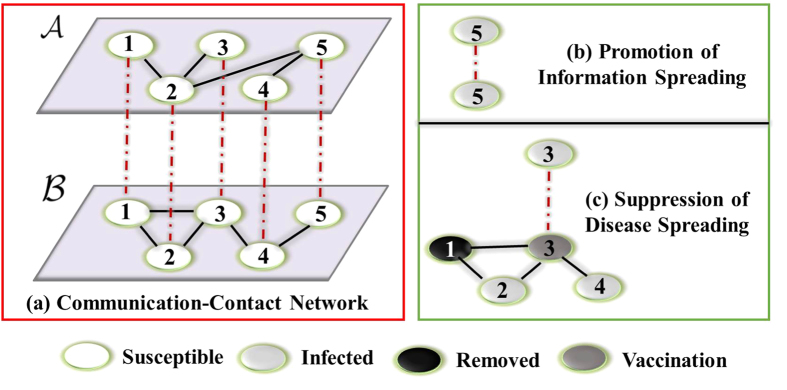
Illustration of asymmetrical mechanisms of information and disease on multiplex networks. (**a**) A multiplex network is used to represent communication and contact networks, which are denoted as layer 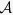 and layer 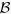, respectively. Each layer has 5 nodes. (**b**) The promotion of information spreading by disease. If node 5 on layer 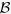 is infected, its counterpart on layer 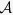 becomes informed. (**c**) The suppression of disease spreading by information diffusion. Node 3 in layer 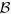 becomes vaccination only when: (1) its counterpart on layer 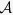 is in the informed state and (2) the number of its infected neighbors on layer 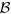 is equal to the threshold *ϕ* = 2.

We generate uncorrelated two-layer networks 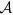 and 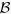 with degree distributions 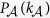 and 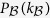, where networks 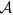 and 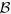 represent the communication and contact networks, respectively. Nodes are individuals and edges are the interactions among individuals. Each node on layer 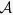 is randomly matched one-to-one with a node of layer 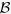. A schematic of the communication-contact coupled networks is shown in Fig. 2(a).

Using the analysis results from real-world data, we construct an asymmetric coevolution information and disease spreading model. In the communication network (layer 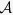) we use the classic susceptible-infected-recovered (SIR) epidemiological model^21^,^34^,^35^ to describe the spreading of information about the disease. Each node can be in one of three states: susceptible, informed, or recovered. A susceptible individual has not acquired any information about the disease, infected (or informed) individuals are aware of the disease and can transmit their information to their neighbors on the communication layer, and recovered individuals have the information but do not transmit it to their neighbors. At each time step, each informed node transmits their information to each susceptible neighbor on layer 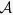 with a probability 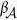. The informed node recovers with a probability 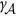. To include the interacting mechanism between information and disease revealed in the real-world data analysis, i.e., that disease spreading promotes the information spreading, we assume that a susceptible node will become informed when its counterpart in layer 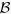 is infected, as shown in Fig. 2(b).

We now introduce a vaccination (V) state into the disease spreading dynamics on the contact network (layer 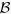) and the model becomes SIRV^36^,^37^. The SIR component of the spreading dynamics is the same as the information spreading on layer 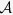 and differs only in the infection and recovery rates, 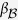 and 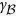, respectively. To introduce the mechanism from our real-world data analysis, i.e., that the spread of information suppresses disease spreading, we assume that an intelligent susceptible individual on layer 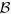 is vaccinated with probability *p* (i) when its counterpart node on layer 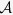 is informed and (ii) when the number of its neighbors in the infected state is equal to or greater than a static threshold *ϕ* [see Fig. 2(c)]. Since immunization is always expensive, condition (i) means that the individual must use the communication network to determine the perniciousness of the disease and condition (ii) means that the individual will adopt immunization measures only when the probability of infection is sufficiently high.

We initiate asymmetrical coupled coevolution dynamics by randomly infecting a tiny fraction of seed nodes on layer 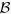 and allowing their counterparts on layer 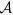 to become informed. We set the effective information transmission and disease transmission rates to be 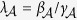 and 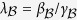, respectively. Without lack of generality we set 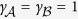. A steady state will be reached when there are no more nodes in the informed or infected state.

### Heterogeneous Mean-field theory

To quantify the asymmetrical coevolution dynamics, we develop a heterogeneous mean-field theory. The outbreak threshold and the fraction of infected or informed nodes in the final state are the two quantities that control the outcome. For the information spreading, the densities of susceptible, informed, and recovered nodes with degree 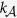 at time *t* are denoted by 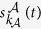, 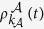, and 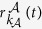, respectively. Analogously, for the disease spreading, the densities of the susceptible, infected, recovered, and vaccinated nodes with degree 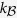 at time *t* are denoted by 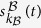, 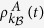, 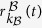, and 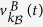, respectively.

We first study the time evolution of information spreading on a communication network, i.e., layer 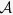. The evolution equation of the susceptible node with degree 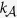 on layer 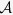 can be written


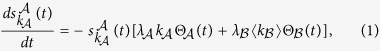


where 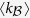 is the average degree of layer 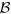, and 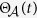 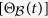 is the probability that a susceptible node connects to an informed (infected) neighbor on uncorrelated layer 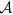 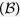 (see details in the Supporting Information). The increase in 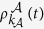 is equal to the decrease in 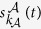, and thus the evolution equations for 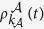 and 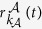 are


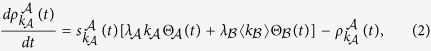


and


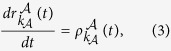


respectively.

We next investigate the evolution of the disease spreading on layer 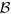, the contact network. The time evolution equations for the susceptible, infected, recovered, and vaccinated nodes on layer 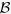 are


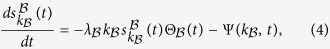



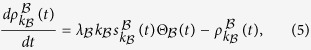



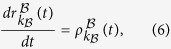


and


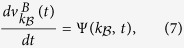


respectively, where 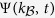 is the probability that a susceptible node on layer 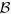 with degree 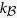 will be vaccinated. More details about the Eqs (1, 2, 3, 4, 5, 6, 7) can be found in the Supporting Information.

We describe the asymmetrical coevolution dynamics of information and disease spreading using Eqs (1, 2, 3) and (4, 5, 6, 7), which allow us to obtain the density of each distinct state on layer 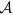 and 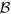 at time *t*, i.e.,


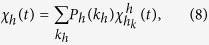


where 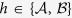 and *χ* ∈ {*S*, *I*, *R*, *V*}. When *t* → ∞, in the steady state, the final sizes of information and disease systems are 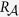 and 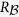, respectively.

Initially only a tiny fraction of nodes on layers 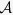 and 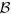 are informed or infected, and most are susceptible. Thus we have 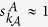, 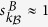. Linearizing Eqs (2) and (5), i.e., neglecting the high order of 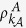 and 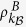, the critical effective information transmission probability is


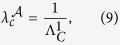


where 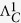 is the maximal eigenvalue of matrix


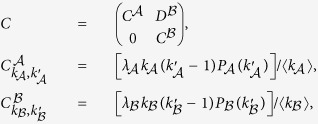


and


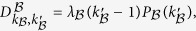


from which we obtain


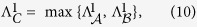


where 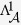 and 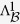 are the maximal eigenvalues of the adjacent matrix of layers 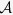 and 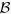, respectively. More details can be found in the Supporting Information. The critical value 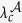 separates information spreading dynamics into local and global information regions. When 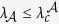, it is in the local information region. When 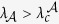, it is in the global information region. In Eq. (9) the global information outbreak condition is correlated only with the topologies of layers 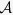 and 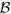, i.e., the immunization probability *p* and threshold *ϕ* do not affect the outbreak of information, but increasing the degree heterogeneity of layers 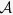 and 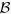 increases the information outbreak probability.

When 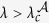, immunization can suppress disease spreading on subnetwork 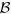, and thus here immunization process and disease spreading can be treated as competing processes^3^. Ref. 3 demonstrates that the two competing processes can be treated as one after the other in the thermodynamic limit. When the immunization process spreads more quickly than the disease, it first spreads on layer 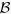 and then the disease spreads on the residual network (i.e., the network after immunization). When the disease spreads more quickly than the immunization, the opposite occurs. Using refs 3 and 17 we find that the immunization progresses more quickly than the disease, i.e., 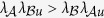, in which 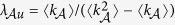 and 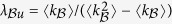, which are the thresholds for the SIR model on a one-layer network^21^, and 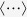 are the moments of the degree distribution. Because in many real-world scenarios information spreads more quickly than disease, we focus on that case. Thus immunization and disease spreading on layer 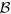 can be treated successively and separately. When *ϕ* = 0, the approximate disease threshold is


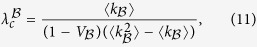


which is the same as in ref. 17. In Eq. (11), where 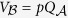, and 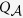 is the final density of the informed population without disease spreading obtained using link percolation theory^21^. From Eq. (11) we can see that, as expected, the threshold is bigger than in the SIR model without vaccination.

When *ϕ* ≥ 1 we use competing percolation theory to obtain the approximate disease threshold. The information first spreads on layer 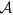, and then the disease spreads on layer 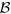. Although many nodes on layer 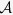 receive the information for large values of 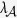, the counterparts of those informed nodes still cannot be immunized when 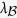 is small. This is the case because according to the proposed model the susceptible nodes that are vaccinated must have authentication from both layers 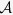 and 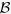. These informed nodes cannot acquire authentication from layer 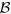 when 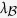 is below the disease threshold. Only for large values of 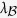, these informed nodes can obtain authentication simultaneously from layers 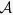 and 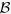. Here the immunized nodes are *V*_*B*_ ≈ 0 and thus the approximate disease threshold is


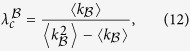


which is the same as the outbreak threshold of SIR disease^21^, i.e., this kind of information-based immunization strategy does not affect the disease outbreak threshold, and this differs from the existing results^16^,^17^. The disease threshold is dependent only on the topology of layer 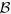 and is independent of the topology of layer 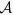, the immunization probability *p*, and the threshold *ϕ*. The asymmetrical coevolution mechanisms presented in our model may explain why the disease threshold is not altered in some real-world situations^38^,^39^,^40^.

### Simulation results

We perform extensive stochastic simulations to study the proposed asymmetrically interacting spreading dynamics on multiplex networks. In the simulations the network sizes and average degrees are set at 
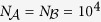
 and 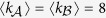, respectively. We use the uncorrelated configuration model to generate layers 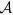 and 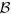 according to the given degree distributions^41^,^42^. For each multiplex network, we perform the dynamics 10^4^ times and measure the average final fraction of information size 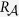, disease size 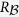, and immunization size 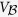 with five randomly selected seeds in layer *B*. We then average these results over 100 network realizations.

To understand the coevolution dynamics of information and disease, we use Erdős-Rényi (ER) networks to represent the communication and contact networks. The degree distributions of layer 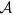 and layer 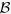 are 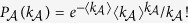 and 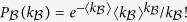, respectively.

**Figure 3:**
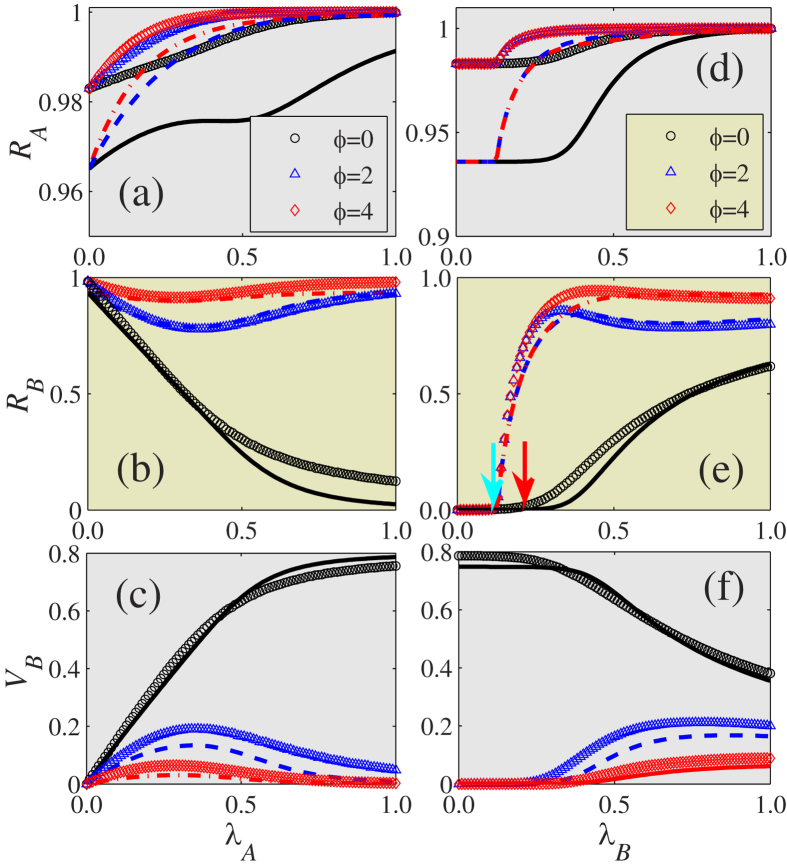
With immunization thresholds *ϕ* being the parameter of interest, the final sizes of information, disease and vaccination on two layer ER-ER multiplex networks. (**a**) The final information size 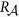, (**b**) the final disease size 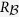, and (**c**) the final vaccination size 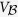 versus information transmission rate 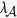 for different values of immunization threshold 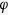 with 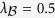. For different values of *ϕ*, (**d**) 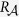, (**e**) 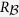 and (**f**) 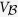 as a function of 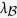 at 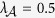. The symbols represent the simulation results and the lines are the theoretical predictions obtained by numerically solving Eqs (1, 2, 3) and (4, 5, 6, 7). In (**e**), the two arrows respectively indicate the numerical disease thresholds for *ϕ* ≥ 1 and *ϕ* = 0, which are obtained by observing *χ*. Other dynamical parameters are set to be 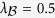 and *p* = 0.8.

Figure 3 shows how the immunization threshold *ϕ* affects the final information, disease, and vaccination sizes. For the information spreading on layer 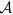, we find that 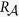 increases with 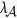 and 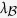 [see Fig. 3(a,d)]. In addition, 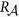 increases with *ϕ* because the individuals in layer 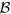 need a large *ϕ* value to guide their immunization decisions [see Fig. 3(c,f)], which causes 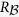 to increase with *ϕ* [see Fig. 3(b,e)]. As a result, the information spreading increases as disease spreading increases.

Figure 3(b,e) show that 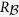 increases with *ϕ*, since individuals are increasingly reluctant to be immunized as *ϕ* increases, and this causes 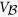 to decrease with *ϕ* [see Fig. 3(c,f)]. Note that 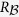 and 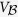 as a function of 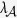 have a non-monotonic shape for *ϕ* = 2 and 4, that 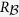 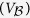 first decreases (increases) with 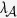 and then increases (decreases) with 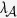. Thus there is an optimal information transmission rate 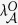 at which 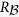 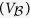 reaches its minimum (maximum) value. Qualitatively this is because a node on layer 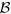 will be immunized only (i) when its counterpart on layer 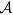 is informed, and (ii) when the number of its infected neighbors 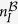 is larger than *ϕ*. For a given 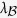, condition (i) is difficult to fulfill when 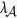 is small and the spread of the information is slow. Increasing 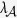 allows more nodes to fulfill condition (i) and allows 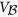 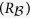 to increase (decrease) with 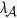. When the value of 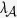 is very large the information spreads so rapidly that condition (ii) can no longer be satisfied. Thus 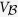 decreases with 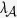, which enhances the spread of disease. The optimal phenomenon is not qualitatively affected by the recovery rates of information and disease. As shown in Fig. 3(e), 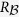 versus 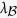 displays a non-monotonic shape for *ϕ* = 2 and 4, i.e., 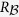 first increases with 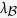 and then decreases. When 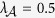 the information spreading is rapid. Increasing 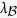 allows more nodes to fulfill the second immunization condition and to be immunized [see Fig. 3(f)], and further leads to the decrease (*ϕ* = 2) or saturation (*ϕ* = 4) of 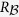 with 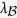. The theoretical predictions of our heterogeneous mean-field theory agree with the simulation predictions. The differences between the theoretical predictions and the simulations are caused by the dynamic correlations among the states of the neighbors and by finite-size network effects^17^. The dynamic correlations are produced when the information (disease) transmission events to one node in layer 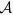 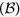 coming from two distinct neighbors are correlated^43^. In the case of coevolution dynamics, the dynamic correlations are also induced by the counterparts of susceptible nodes^4^.

For the disease spreading on layer 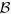, the disease threshold 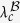 for *ϕ* = 0 is clearly larger than the threshold 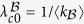, which is the disease threshold without immunization (i.e., *p* = 0) [see the right arrow in Fig. 3(e)]. We can determine the numerical disease threshold by measuring the susceptibility^44^ or variability^45^ (see details in Method). Note that the disease threshold 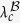 for *ϕ* ≥ 1 is the same as 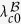, which is consistent with the theoretical prediction [see Eq. (12) and the left arrow in Fig. 3(e)]. This occurs because individuals choose immunization only when the number of their infected neighbors is equal to or greater than *ϕ*. The asymmetrical coevolution mechanisms proposed in our model may explain why choosing to be immunized during disease spreading does not affect the disease threshold^38^,^39^,^40^.

**Figure 4:**
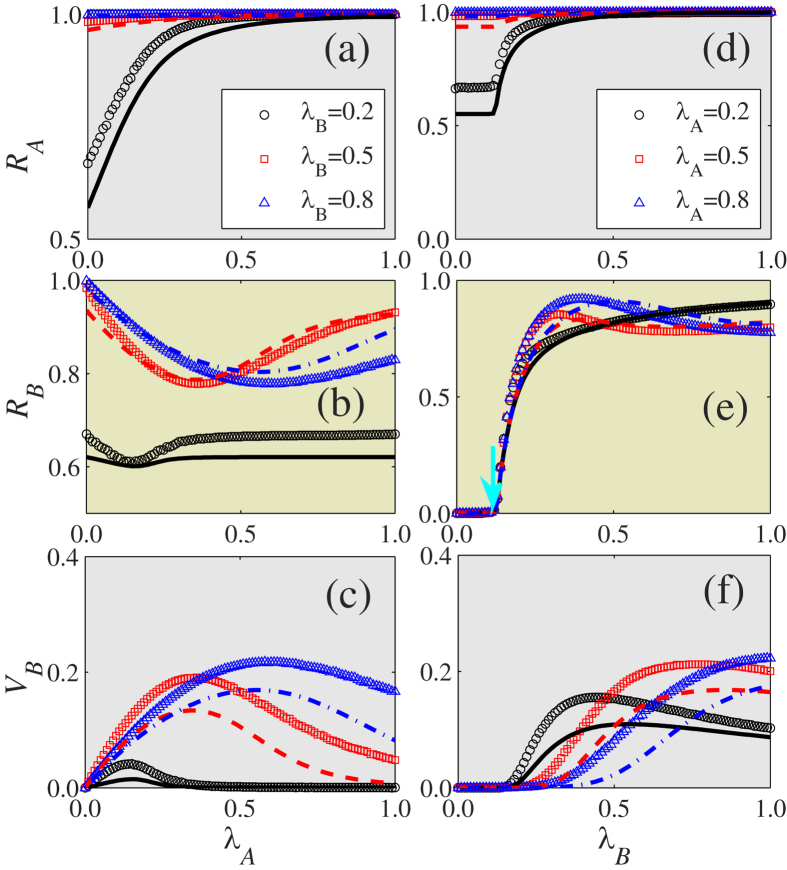
With disease transmission rate 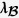 being the parameter of interest, the asymmetrically interacting dynamics spreads on ER-ER networks. (**a**) The final information size 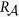, (**b**) the final disease size 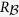, and (**c**) the vaccination size 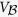 versus the information transmission rate 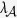 for the disease transmission rate 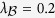, 0.5 and 0.8. For 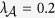, 0.5 and 0.8, (**d**) 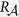, (**e**) 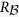 and (**f**) 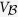 as a function of 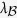. In the figures, symbols are the simulation results and the lines are the theoretical predictions. In (**e**), the arrow indicates the numerical disease threshold. We set other parameters to be *ϕ* = 2 and *p* = 0.8.

We use *ϕ* = 2 to measure the final information and disease sizes (see Fig. 4). According to Eq. (12), the disease threshold is 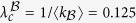. When 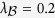, 0.5, and 0.8, any value of 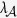 can cause an information outbreak due to an outbreak of disease on layer 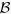 [see Fig. 4(a)]. Thus the information outbreak threshold 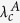 is zero. Figure 4(b,c) show the optimal information transmission rate 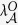 at which 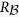 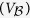 reaches its minimum (maximum) value. When 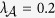, 0.5, and 0.8, 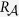 increases with 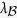 because of the increase in the disease [see Fig. 4(d)]. Note that 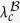 is not affected by 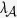 [see the arrow in Fig. 4(e)]. As shown in Fig. 4(e), 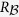 versus 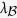 first increases and then decreases for large 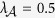 and 0.8. This phenomenon can be understood in the same way with Fig. 3(e). There is again good agreement between the theoretical and numerical results.

**Figure 5:**
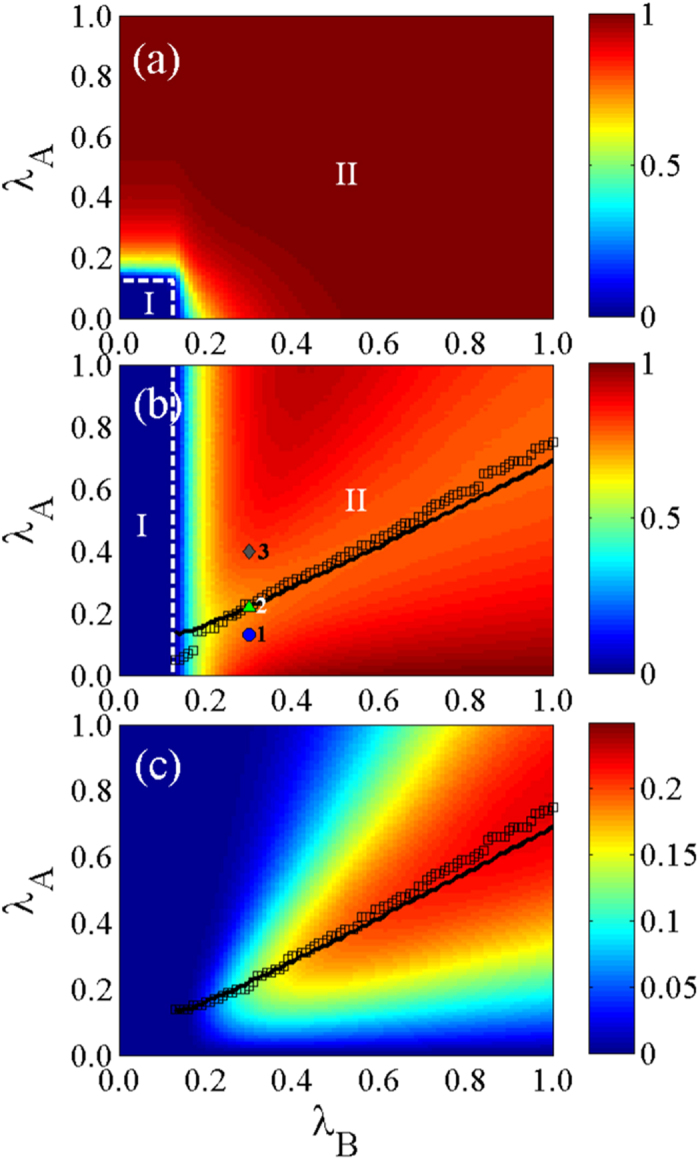
Asymmetrically interacting dynamics on ER-ER networks. The final density in each state relating the parameters 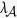 and 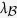: (**a**) the final information size 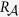, (**b**) the final disease size 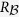 and (c) the vaccination size 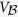. In (**a**), the horizontal and vertical dashed lines separate the 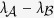 plane into local and global information outbreak regions, which are denoted as regions I and II. In (**b**), the vertical dashed line divides the plane into a local (region I) and a global (region II) disease outbreak regions. In (**b**), the blue circles (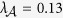, 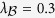), green up triangle (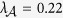, 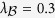) and gray diamond (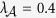, 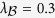) represent 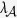 being below, at and above 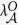, respectively (see more discussions in Fig. 6). The black squares (black lines) in (**b**,**c**) represent the optimal information transmission rate 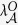 versus 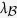. Other parameters are set to be *ϕ* = 2 and *p* = 0.8.

Figure 5 shows the effects of 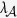 and 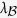 on the final steady state for *R*_*A*_, *R*_*B*_, and *V*_*B*_ for *ϕ* = 2 and shows the phase diagrams for the final sizes as a function of *λ*_*A*_ and *λ*_*B*_. Figure 5(a) shows that 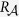 increases with 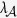 and 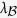. The 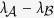 plane is divided into a local (I) and global (II) information outbreak regions. In Fig. 5(a) region I and region II are separated by the 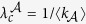 (horizontal white dashed line) and 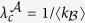 (vertical white dashed line) obtained from Eq. (10). Figure 5(b) shows how region I and region II are separated by 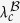 (see vertical white dashed line). For the minimum value of 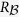 in region II, 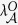 increases linearly with 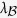, as shown in Fig. 5(b) [see black lines and symbols in (b,c)]. At the optimal 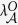, 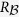 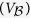 reaches its minimum (maximum) value, as shown in Fig. 5(b,c). Note that 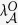 is slightly smaller than 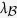 because whether information induces an individual to be vaccinated depends on the infection level of their neighbors. Our heterogeneous mean-field theory describes this phenomenon very well.

**Figure 6:**
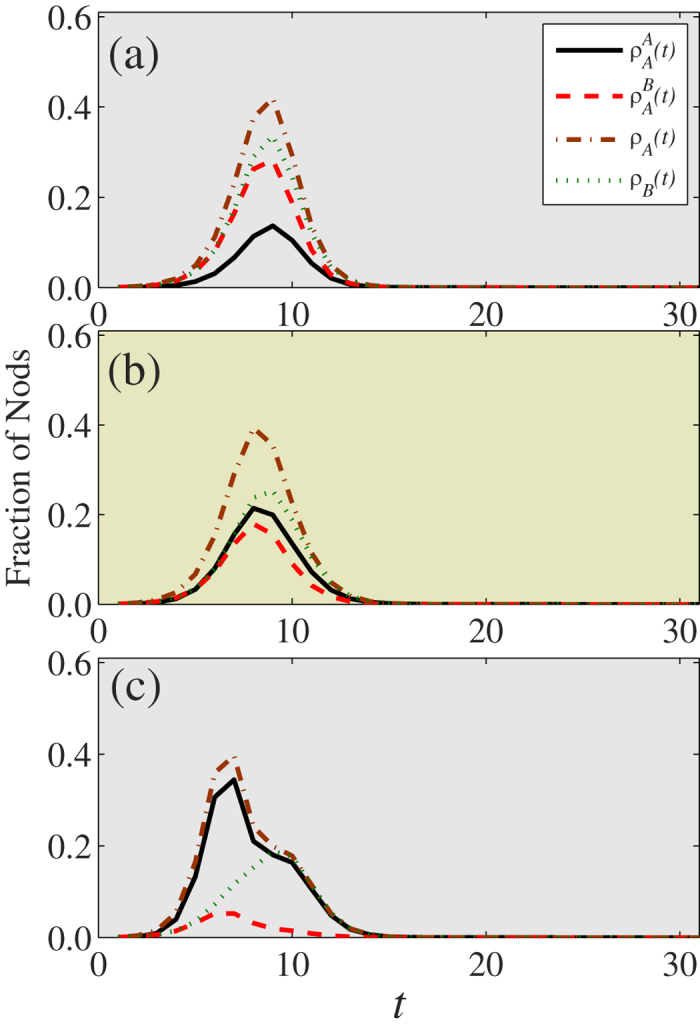
On ER-ER coupled networks, the time evolution of each type of nodes. The time evolution of 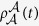, 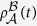, 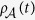 and 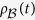 for (**a**) 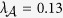, (**b**) 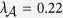 and (**c**) 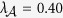. Other parameters are set to be 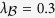, *ϕ* = 2 and *p* = 0.8.

Thus we know that for a given disease transmission rate there is an optimal information transmission rate at which the disease spreading is markedly reduced. In order to determine the coevolution characteristics of information and disease spreading when the information reaches its optimal transmission, we first look at the macroscopic coevolution of the two dynamics under different information transmission rates as shown in Fig. 6. We denote the fraction of nodes on layer 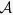 informed by their neighbors or by their counterpart nodes using 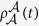 and 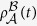, respectively. Here 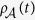 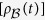 is the fraction of nodes obtaining the information (disease) on layer 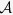 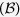 at time *t*. For small 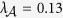 below 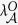 [see Fig. 6(a)], 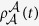, 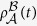, and 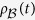 reach their peaks simultaneously. Note that 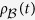 is larger than 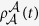 and very close to 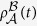, which means that the spread of information is primarily induced by the disease outbreak. At 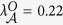, we find that 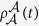, 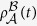, and 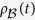 reach their peaks simultaneously, and that 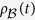 is closer to 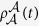 than to 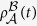. Thus the information and disease have a similar spreading velocity. For a large value of 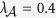, the information spreads more quickly than the disease. Our results suggest that information and disease spreading have a similar macroscopic coevolution characteristic when the information transmission rate is at its optimal value.

**Figure 7:**
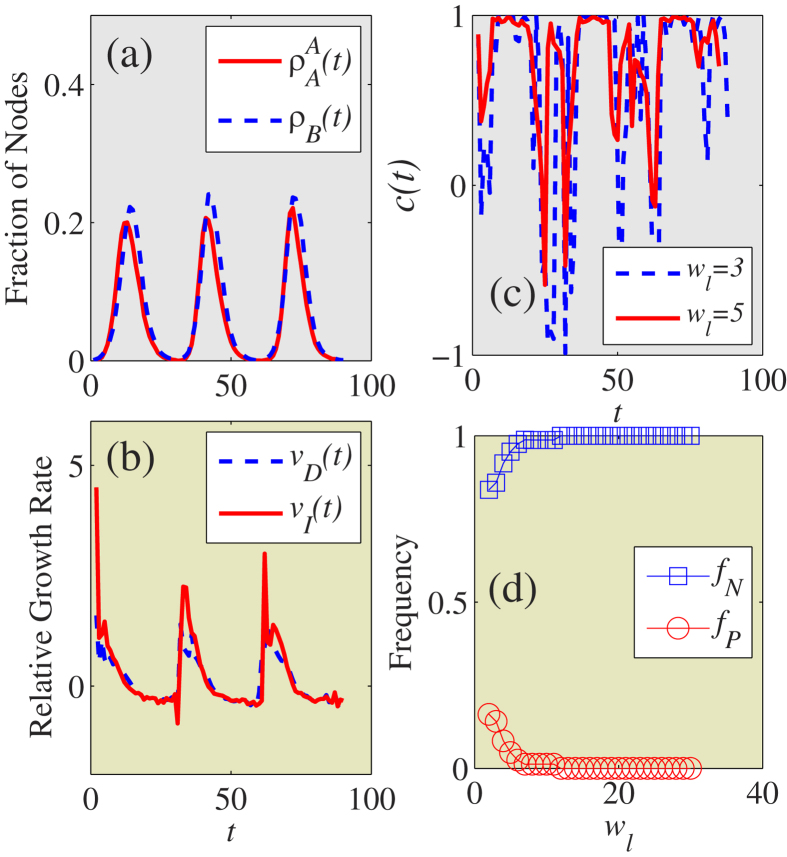
Asymmetrically interacting spreading dynamics on coupled ER-ER networks at the optimal information transmission rate. (**a**) The fractions of nodes in the informed state 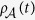 (red solid line) and infected state 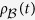 (blue dashed line) versus *t*. (**b**) The relative growth rates *v*_*D*_(*t*) (blue dashed line) and *v*_*I*_(*t*) (red solid line) of 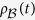 and 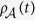 versus *t*, respectively. (**c**) Cross-correlations *c*(*t*) between *v*_*I*_(*t*) and *v*_*D*_(*t*) for the given window size *w*_*l*_ = 3 (blue dashed line) and *w*_*l*_ = 5 (red solid line). (**d**) The fractions of negative correlations *f*_*P*_ (blue squares) and positive correlations *f*_*N*_ (red circles) as a function of *w*_*I*_. We set other parameters to be 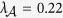, 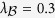 and *p* = 0.8, respectively.

Figure 7 shows the microscopic coevolution characteristics of the two dynamics at the optimal information transmission rate. Figure 7(a) shows the time evolution of information and disease in three independent dynamical realizations that have similar trends in their macroscopic coevolution of information spreading and disease spreading. Figure 7(b) shows the relative growth rates of information *v*_*I*_(*t*) and disease *v*_*D*_(*t*). As in the real-world case in Fig. 1(b), the same and opposite growth trends are observed. Figure 7(c) shows the calculated cross-correlations between the two time series of *v*_*D*_(*t*) and *v*_*I*_(*t*). Both positive and negative cross-correlations exist when the window size is small [see Fig. 7(d)]. Note that Fig. 7 agrees well with the real-world situation shown in Fig. 1. Through extensive simulations, we find that heterogeneous networks display a similar phenomenon. Thus the coevolution between information and disease can become optimal in which the macroscopic and microscopic coevolution characteristics of information and disease exhibit similar trends and the information diffusion greatly suppresses the spread of disease.

**Figure 8:**
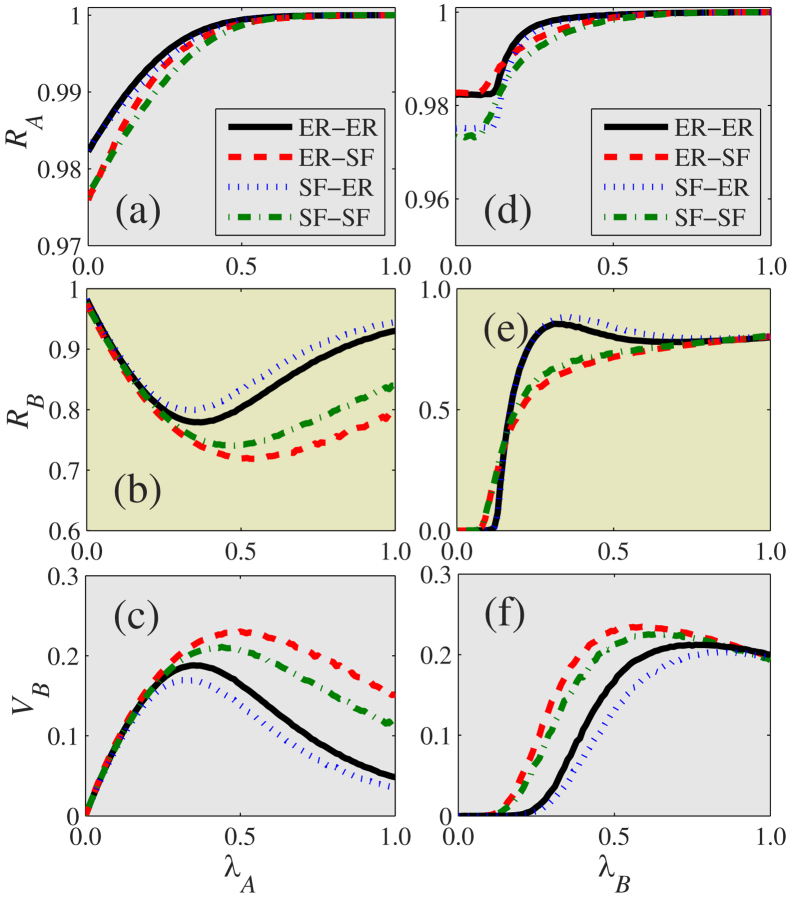
Effect of degree heterogeneity on coevolution dynamics. (**a**) The final information size 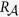, (**b**) the final disease size 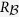 and (**c**) the vaccination size 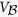 versus the information transmission rate 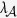 on ER-ER, ER-SF, SF-ER and SF-SF coupled networks with 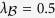. For ER-ER, ER-SF, SF-ER and SF-SF networks with 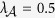, (**d**) 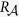, (**e**) 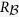 and (**f**) 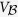 as a function of 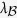. Other parameters are set to be *ϕ* = 2, *p* = 0.8 and 〈*k*_*A*_〉 = 〈*k*_*B*_〉 = 8.

To examine how topology affects multiplex systems, we next simulate different possible heterogeneities in the communication and contact networks (see Fig. 8). We generate scale-free (SF) networks with a power-law degree distribution 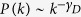 by using an uncorrelated configuration model^41^,^46^ in which *γ*_*D*_ is the degree exponent. Through extensive simulations we find that the values of *γ*_*D*_ do not qualitatively affect the results. Without loss of generality we set *γ*_*D*_ = 3.0. Note that there is an optimal information transmission rate at which the disease is significantly suppressed [see Fig. 8(b,c)], and thus heterogeneity in network topology does not qualitatively affect this optimal phenomenon. We also find that the multiplex networks with a homogeneous communication layer and a heterogeneous contact layer have a greater optimal information transmission rate. As the information (disease) spreads more (less) widely on homogeneous (heterogeneous) networks for a large transmission rate, 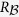 is further reduced. Figure 8(e) shows that the disease threshold 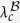 is determined only by the topology of layer 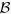, and that the topology of layer 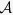 does not affect 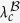.

For information spreading on layer 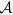 as shown in Fig. 8(a), 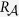 decreases with the degree heterogeneity of layer 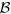, since a homogeneous contact network facilitates the spread of disease for large 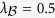
20. In Fig. 8(b,c), the effects of the heterogeneity of layer 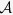 on 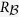 and 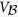 are negligible when 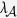 is small, but 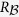 increases with the heterogeneity of layer 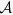 when 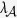 is large because it is more difficult to immunize nodes [i.e., 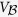 decreases with the heterogeneity of layer 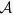 in Fig. 8(c)].

Figure 8(d–f) show 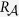, 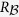 and 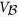 as a function of *λ*_*B*_ on several networks for large 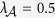. The degree heterogeneity of layer 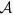 is a factor. When 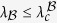, 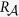 decreases with the heterogeneity of layer 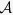, but the effects of the heterogeneity of layer 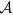 on 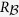 and 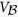 are negligible. When 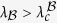 the heterogeneity of layer 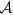 does not increase information diffusion, but promotes disease spreading because nodes are less likely to be immunized. We examine the effects of the heterogeneity of layer 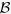 and find that 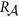 and 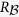 increase (decrease) with the degree heterogeneity of layer 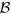 for small (large) 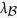. When the degree heterogeneity of layer 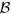 is increased, the network has a large number of individuals with very small degrees and more individuals with large degrees. When 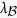 is small there are more hubs in heterogeneous networks that facilitate disease spreading because they are more likely to be infected, and this increases information diffusion. When 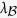 is large, however, there are many small-degree nodes with a low probability of being infected, and this produces smaller values of 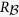, which causes smaller values of 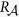.

## Discussion

We have systematically investigated the coevolution dynamics of information and disease spreading on multiplex networks. We first discover indications of asymmetrical interactions between the two spreading dynamics by analyzing real data, i.e., the weekly time series of information spreading and disease spreading in the form of influenza-like illness (ILI) evolving simultaneously in the US during an approximate 200-week period from 3 January 2010 to 10 December 2013. Using these interacting mechanisms observed in real data, we propose a mathematical model for describing the coevolution spreading dynamics of information and disease on multiplex networks. We investigate the coupled dynamics using heterogeneous mean-field theory and stochastic simulations. We find that information outbreaks can be triggered by the spreading dynamics within a communications network and also by disease outbreaks in the disease contact network, but we also find that the disease threshold is not affected by information spreading, i.e., that the outbreak of disease is solely dependent on the topology of the contact network. More important, for a given rate of disease transmission we find that there is an optimal information transmission rate that decreases the disease size to a minimum value, and the modeled evolution of information and disease spreading is consistent with real-world behavior. We also verify that heterogeneity in network topology does not invalidate the results. In addition, we find that when information diffuses slowly, the degree heterogeneity of the communication network has a trivial impact on disease spreading. The homogeneity of the communication network can enhance the vaccination size and thus prevent disease spreading more effectively when the spread of information is rapid.

The asymmetrical interacting mechanism we discover by analyzing real-world data provides solid evidence supporting the basic assumptions of previous researches^16^,^17^. Our data-driven model also reveals some fundamental coevolution mechanisms in the coevolution dynamics. Using these coevolution dynamics of information and disease we are able to identify phenomena that differ qualitatively from those found in previous research on disease-behavior systems. Our results enable us to quantify the optimal level of information transmission that suppresses disease spreading. The coevolution mechanisms also enable us to better understand why the disease threshold is unchanged even when information spreading in some real-world situations undergoes coevolution.

Further research on disease-behavior systems promises to discover additional real-world mechanisms that can be used to refine models of coevolution spreading dynamics. Developing a more accurate theoretical method is full of challenges because it is difficult to describe the strong dynamic correlations among the states of neighboring nodes in a network. If we take dynamical correlations into account, we may be able to use such advanced theoretical methods as dynamic message-passing^47^,^48^ or pair approximation^49^,^50^.

## Methods

### Relative growth rates

We define the relative growth rates *v*_*G*_(*t*) of *n*_*G*_(*t*) and *v*_*D*_(*t*) of *n*_*D*_(*t*) to be


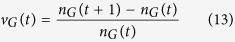


and


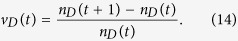


If *v*_*G*_(*t*) > 0 [*v*_*D*_(*t*) > 0], *n*_*G*_(*t*) [*n*_*D*_(*t*)] shows an increasing trend at time *t*. If not, *n*_*G*_(*t*) [*n*_*D*_(*t*)] shows a decreasing trend at time *t*.

### Variability measure

The variability *χ*^42^,^45^ is


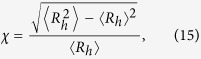


where *R*_*h*_ is the final information size 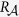 or disease size 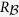, and 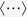 is the ensemble averaging. The value of *χ* exhibits a peak at the critical point at which the thresholds can be computed.

## Additional Information

**How to cite this article**: Wang, W. *et al*. Suppressing disease spreading by using information diffusion on multiplex networks. *Sci. Rep.* **6**, 29259; doi: 10.1038/srep29259 (2016).

## Supplementary Material

Supporting Information
