# Supplementary material for: Suppressing disease spreading by using information diffusion on multiplex networks
Source: Sci Rep. 2016 Jul 6;6:29259. doi: 10.1038/srep29259 (PMC4933956; doi:10.1038/srep29259)
Supplement: Supporting Information [file srep29259-s1.pdf]

Supporting information for 'Suppressing disease spreading by using information diffusion on multiplex networks'

Wei Wang,[1,2,3] Quan-Hui Liu,[1,2] Shi-Min Cai,[1,2] Ming Tang,[1,2,*] Lidia A. Braunstein,[3,4] and H. Eugene Stanley[3]

[1] *Web Sciences Center, University of Electronic Science and Technology of China, Chengdu 610054, China*
[2] *Big Data Research Center, University of Electronic Science and Technology of China, Chengdu 610054, China*
[3] *Center for Polymer Studies and Department of Physics, Boston University, Boston, Massachusetts 02215, USA*
[4] *Instituto de Investigaciones Físicas de Mar del Plata (IFIMAR)-Departamento de Física, Facultad de Ciencias Exactas y Naturales, Universidad Nacional de Mar del Plata-CONICET, Funes 3350, (7600) Mar del Plata, Argentina.*

(Dated: May 24, 2016)

PACS numbers: 89.75.Hc, 87.19.X-, 87.23.Ge

I. HETEROGENEOUS MEAN-FIELD THEORY

To quantify the asymmetrical coevolution dynamics, we develop a heterogeneous mean-field theory. The outbreak threshold and the fraction of infected or informed nodes in the final state are the two quantities that control the outcome. For the information spreading, the densities of susceptible, informed, and recovered nodes with degree $k_{\mathcal{A}}$ at time t are denoted by $s^{\mathcal{A}}_{k_{\mathcal{A}}}(t)$, $\rho^{\mathcal{A}}_{k_{\mathcal{A}}}(t)$, $r^{\mathcal{A}}_{k_{\mathcal{A}}}(t)$, respectively. Analogously, for the disease spreading, the densities of the susceptible, infected, recovered and vaccinated nodes with degree $k_{\mathcal{B}}$ at time t are denoted by $s^{\mathcal{B}}_{k_{\mathcal{B}}}(t)$, $\rho^{\mathcal{A}}_{k_{\mathcal{B}}}(t)$, $r^{\mathcal{B}}_{k_{\mathcal{B}}}(t)$ and $v^{\mathcal{B}}_{k_{\mathcal{B}}}(t)$, respectively.

We first study the time evolution of information spreading on a communication network, i.e., layer $\mathcal{A}$. During a short period of time $[t, t+dt]$ there are two ways for a susceptible node denoted by $u_{\mathcal{A}}$ with degree $k_{\mathcal{A}}$ to become informed, (i) node $u_{\mathcal{A}}$ can acquire the information from its neighbors with a probability $\lambda_{\mathcal{A}} k_{\mathcal{A}} \Theta_{\mathcal{A}}(t)dt$, where $\Theta_{\mathcal{A}}(t)$ is the probability that a susceptible node connects to an informed neighbor on uncorrelated layer $\mathcal{A}$, which is given by [1]

$$\Theta_{\mathcal{A}}(t) = \frac{1}{\langle k_{\mathcal{A}}\rangle} \sum_{k'_{\mathcal{A}}} (k'_{\mathcal{A}} - 1) P_{\mathcal{A}}(k'_{\mathcal{A}}) \rho^{\mathcal{A}}_{k'_{\mathcal{A}}}(t), \tag{1}$$

where $\langle k_{\mathcal{A}}\rangle$ is the average degree of layer $\mathcal{A}$, or (ii) node $u_{\mathcal{A}}$ can acquire the information when its counterpart node $u_{\mathcal{B}}$ on layer $\mathcal{B}$ becomes infected. From a mean-field perspective, node $u_{\mathcal{A}}$ acquires the information from its counterpart node with a probability $\lambda_{\mathcal{B}} \sum_{k_{\mathcal{B}}} P_{\mathcal{B}}(k_{\mathcal{B}}) k_{\mathcal{B}} \Theta_{\mathcal{B}}(t)dt$, where $\Theta_{\mathcal{B}}(t)$ (to be defined later) is the probability that a susceptible node on layer $\mathcal{B}$ will connect to an infected neighbor. Taking the above two cases into consideration, the evolution equation of the susceptible node with degree $k_{\mathcal{A}}$ on layer $\mathcal{A}$ can be written as

$$\frac{ds^{\mathcal{A}}_{k_{\mathcal{A}}}(t)}{dt} = -s^{\mathcal{A}}_{k_{\mathcal{A}}}(t)[\lambda_{\mathcal{A}} k_{\mathcal{A}} \Theta_{\mathcal{A}}(t) + \lambda_{\mathcal{B}} \langle k_{\mathcal{B}}\rangle \Theta_{\mathcal{B}}(t)]. \tag{2}$$

The increase in $\rho^{\mathcal{A}}_{k_{\mathcal{A}}}(t)$ is equal to the decrease in $s^{\mathcal{A}}_{k_{\mathcal{A}}}(t)$, and thus the evolution equations for $\rho^{\mathcal{A}}_{k_{\mathcal{A}}}(t)$ and $r^{\mathcal{A}}_{k_{\mathcal{A}}}(t)$ are

$$\frac{d\rho^{\mathcal{A}}_{k_{\mathcal{A}}}(t)}{dt} = s^{\mathcal{A}}_{k_{\mathcal{A}}}(t)[\lambda_{\mathcal{A}} k_{\mathcal{A}} \Theta_{\mathcal{A}}(t) + \lambda_{\mathcal{B}} \langle k_{\mathcal{B}}\rangle \Theta_{\mathcal{B}}(t)] - \rho^{\mathcal{A}}_{k_{\mathcal{A}}}(t), \tag{3}$$

and

$$\frac{dr^{\mathcal{A}}_{k_{\mathcal{A}}}(t)}{dt} = \rho^{\mathcal{A}}_{k_{\mathcal{A}}}(t), \tag{4}$$

respectively.

We next investigate the evolution of the disease spreading on layer $\mathcal{B}$, the contact network. During a short time period $[t, t+dt]$, a susceptible node $u_{\mathcal{B}}$ with degree $k_{\mathcal{B}}$ in layer $\mathcal{B}$ can either be infected or vaccinated. The probability of node $u_{\mathcal{B}}$ being infected is $\lambda_{\mathcal{B}} k_{\mathcal{B}} \Theta_{\mathcal{B}}(t)dt$, where

$$\Theta_{\mathcal{B}}(t) = \frac{1}{\langle k_{\mathcal{B}}\rangle} \sum_{k'_{\mathcal{B}}} (k'_{\mathcal{B}} - 1) P_{\mathcal{B}}(k'_{\mathcal{B}}) \rho^{\mathcal{B}}_{k'_{\mathcal{B}}}(t), \tag{5}$$

where $\langle k_{\mathcal{B}}\rangle$ is the average degree of layer. Node $u_{\mathcal{B}}$ can be vaccinated only when (1) its counterpart node $u_{\mathcal{A}}$ is in the informed state and (2) it is aware of the danger on layer $\mathcal{B}$, i.e., the number of infected neighbors $n^{\mathcal{B}}_I$ does not fall below static threshold ϕ. Since a node in layer $\mathcal{A}$ can be susceptible only when its counterpart is susceptible, node $u_{\mathcal{B}}$ meets the first condition with probability $\sum_{k_{\mathcal{A}}} P_{\mathcal{A}}(k_{\mathcal{A}}) s^{\mathcal{A}}_{k_{\mathcal{A}}} k_{\mathcal{A}} \lambda_{\mathcal{A}} \Theta_{\mathcal{A}}(t)$. The probability of the second immunization condition for node $u_{\mathcal{B}}$ is

$$\Upsilon(k_{\mathcal{B}}, t) = \sum_{n^{\mathcal{B}}_I = \phi}^{k_{\mathcal{B}}} \Omega(k_{\mathcal{B}}, n^{\mathcal{B}}_I, t), \tag{6}$$

where $\Omega(k_{\mathcal{B}}, n^{\mathcal{B}}_I, t)$ is the probability that a susceptible node on layer $\mathcal{B}$ with degree $k_{\mathcal{B}}$ has $n^{\mathcal{B}}_I$ infected neighbors, which can be expressed as

$$\Omega(k_{\mathcal{B}}, n^{\mathcal{B}}_I, t) = \binom{k_{\mathcal{B}}}{n^{\mathcal{B}}_I} [\Theta_{\mathcal{B}}(t)]^{n^{\mathcal{B}}_I} [1 - \Theta_{\mathcal{B}}(t)]^{k_{\mathcal{B}} - n^{\mathcal{B}}_I}. \tag{7}$$

When node $u_{\mathcal{B}}$ simultaneously fulfills both conditions, it will be vaccinated with probability p. Thus the probability that a susceptible node on layer $\mathcal{B}$ with degree $k_{\mathcal{B}}$ will be vaccinated is

$$\Psi(k_{\mathcal{B}}, t) = p \Upsilon(k_{\mathcal{B}}, t) \sum_{k_{\mathcal{A}}} P_{\mathcal{A}}(k_{\mathcal{A}}) s^{\mathcal{A}}_{k_{\mathcal{A}}} k_{\mathcal{A}} \lambda_{\mathcal{A}} \Theta_{\mathcal{A}}(t). \tag{8}$$

* tangminghan007@gmail.com

We can use these analyses to obtain the time evolution equations for the susceptible, infected, recovered, and vaccinated nodes on layer $\mathcal{B}$, i.e.,

$$\frac{ds^{\mathcal{B}}_{k_{\mathcal{B}}}(t)}{dt} = -\lambda_{\mathcal{B}} k_{\mathcal{B}} s^{\mathcal{B}}_{k_{\mathcal{B}}}(t)\Theta_{\mathcal{B}}(t) - \Psi(k_{\mathcal{B}}, t), \quad (9)$$

$$\frac{d\rho^{\mathcal{B}}_{k_{\mathcal{B}}}(t)}{dt} = \lambda_{\mathcal{B}} k_{\mathcal{B}} s^{\mathcal{B}}_{k_{\mathcal{B}}}(t)\Theta_{\mathcal{B}}(t) - \rho^{\mathcal{B}}_{k_{\mathcal{B}}}(t), \quad (10)$$

$$\frac{dr^{\mathcal{B}}_{k_{\mathcal{B}}}(t)}{dt} = \rho^{\mathcal{B}}_{k_{\mathcal{B}}}(t), \quad (11)$$

and

$$\frac{dv^{B}_{k_{\mathcal{B}}}(t)}{dt} = \Psi(k_{\mathcal{B}}, t), \quad (12)$$

respectively.

We can describe the asymmetrical coevolution dynamics of information and disease spreading using Eqs. (2)-(4) and (9)-(12), which allow us to obtain the density of each distinct state on layer $\mathcal{A}$ and $\mathcal{B}$ at time t, i.e.,

$$\chi_h(t) = \sum_{k_h} P_h(k_h)\chi^h_{h_k}(t), \quad (13)$$

where $h \in \{\mathcal{A}, \mathcal{B}\}$ and $\chi \in \{S, I, R, V\}$. When $t \to \infty$, in the steady state, the final sizes of information and disease systems are $R_{\mathcal{A}}$ and $R_{\mathcal{B}}$, respectively.

II. LINEAR ANALYSIS FOR THE INFORMATION THRESHOLD

Initially only a tiny fraction of nodes on layers $\mathcal{A}$ and $\mathcal{B}$ are informed or infected, and most are susceptible. Thus we have $s^{A}_{k_{\mathcal{A}}} \approx 1$, $s^{B}_{k_{\mathcal{B}}} \approx 1$. Linearizing Eqs. (3) and (10), we have

$$\frac{d\rho^{\mathcal{A}}_{k_{\mathcal{A}}}(t)}{dt} = \lambda_{\mathcal{A}} k_{\mathcal{A}} \Theta_{A}(t) + \lambda_{\mathcal{B}} \langle k_{\mathcal{B}}\rangle \Theta_{B}(t) - \rho^{\mathcal{A}}_{k_{\mathcal{A}}}(t),$$
$$\frac{d\rho^{\mathcal{B}}_{k_{\mathcal{B}}}(t)}{dt} = \lambda_{\mathcal{B}} k_{\mathcal{B}} \Theta_{\mathcal{B}}(t) - \rho^{\mathcal{B}}_{k_{\mathcal{B}}}(t). \quad (14)$$

We rewrite Eq. (14) in matrix form as

$$\frac{d\vec{\rho}}{dt} = C\vec{\rho} - \vec{\rho}, \quad (15)$$

where

$$\vec{\rho} \equiv (\rho^{\mathcal{A}}_{k_{\mathcal{A}}=1}, \dots, \rho^{\mathcal{A}}_{k_{\mathcal{A},max}}, \rho^{\mathcal{B}}_{k_{\mathcal{B}}=1}, \dots, \rho^{\mathcal{B}}_{k_{\mathcal{B},max}})^T,$$

and

$$C = \begin{pmatrix} C^{\mathcal{A}} & D^{\mathcal{B}} \\ 0 & C^{\mathcal{B}} \end{pmatrix}. \quad (16)$$

The elements in matrix C are

$$C^{\mathcal{A}}_{k_{\mathcal{A}},k'_{\mathcal{A}}} = [\lambda_{\mathcal{A}} k_{\mathcal{A}}(k'_{\mathcal{A}} - 1)P_{\mathcal{A}}(k'_{\mathcal{A}})]/\langle k_{\mathcal{A}}\rangle,$$
$$C^{\mathcal{B}}_{k_{\mathcal{B}},k'_{\mathcal{B}}} = [\lambda_{\mathcal{B}} k_{\mathcal{B}}(k'_{\mathcal{B}} - 1)P_{\mathcal{B}}(k'_{\mathcal{B}})]/\langle k_{\mathcal{B}}\rangle,$$

and

$$D^{\mathcal{B}}_{k_{\mathcal{B}},k'_{\mathcal{B}}} = \lambda_{\mathcal{B}}(k'_{\mathcal{B}} - 1)P_{\mathcal{B}}(k'_{\mathcal{B}}).$$

If $\rho_A(t)$ increases exponentially at the initial time, a finite fraction of nodes on layer $\mathcal{A}$ will receive information. If it does not, only a tiny fraction of nodes will receive information. Thus the critical effective information transmission probability is

$$\lambda^{\mathcal{A}}_c = \frac{1}{\Lambda^1_C}, \quad (17)$$

where Λ^1_C is the maximal eigenvalue of matrix C, from which we obtain

$$\Lambda^1_C = \max\{\Lambda^1_{\mathcal{A}}, \Lambda^1_{\mathcal{B}}\}, \quad (18)$$

where $\Lambda^1_{\mathcal{A}}$ and $\Lambda^1_{\mathcal{B}}$ are the maximal eigenvalues of the adjacent matrix of layers $\mathcal{A}$ and $\mathcal{B}$, respectively.

[1] Newman, M. E. J. Networks An Introduction (Oxford University Press, Oxford, 2010).
